# Unsuccessful introduced biocontrol agents can act as pollinators of invasive weeds: Bitou Bush (*Chrysanthemoides monilifera* ssp. *rotundata*) as an example

**DOI:** 10.1002/ece3.3441

**Published:** 2017-09-21

**Authors:** Caroline L. Gross, Joshua D. Whitehead, Camila Silveira de Souza, David Mackay

**Affiliations:** ^1^ Ecosystem Management University of New England Armidale NSW Australia; ^2^ Programa de pós Graduação em Ecologia e Conservação UFMS Campo Grande Brazil

**Keywords:** alien pollinators, alien species, Asteraceae, biocontrol agent, Bitou Seedfly, breeding system, nursery pollinators, self‐incompatibility, weed pollination

## Abstract

The extent of self‐compatibility and reliance on pollinators for seed set are critical determinants of reproductive success in invasive plant species. Seed herbivores are commonly used as biocontrol agents but may also act as flower visitors, potentially resulting in pollination. However, such contrasting or potentially counterproductive interaction effects are rarely considered or evaluated for biological control programs. We investigated the breeding system and pollinators of Bitou Bush (*Chrysanthemoides monilifera* ssp. *rotundata*), an invasive species in Australia that has been the subject of biocontrol programs since 1987. We found the species to be obligate outcrossing in all six populations tested. From 150 video hours, we found 21 species of potential pollinators, including *Mesoclanis polana*, the Bitou Seedfly, native to South Africa and released in Australia as a biocontrol agent in 1996. *Mesoclanis polana* transferred pollen to stigmas and was the most common pollinator (52% of pollinator visits), followed by the syrphid fly *Simosyrphus grandicornis* (9%) and introduced honeybee, *Apis mellifera* (6.5%). Fruit‐to‐flower ratios ranged from 0.12 to 0.45 and were highest in the population with the greatest proportion of *Mesoclanis polana* visits. In an experimental trial, outside the naturalized range, the native bee *Homalictus sphecodoides* and the native syrphid *Melangyna viridiceps* were the primary pollinators, and fruit‐to‐flower ratios were 0.35, indicating that Bitou Bush would have ready pollinators if its range expanded inland. *Synthesis*. Invasive Bitou Bush requires pollinators, and this is effected by a range of generalist pollinators in eastern Australia including the Bitou Seedfly, introduced as a biocontrol agent, and the major pollinator detected in this study. Fruit‐to‐flower ratios were highest when the Bitou Seedfly was in high abundance. This study underscores the importance of evaluating the pollination biology of invasive species in their native ranges and prior to the introduction of biocontrol agents.

## 1 INTRODUCTION

It is estimated that more than 85% of the world's flora requires or benefits from pollen delivery by pollinators (Ollerton, Winfree, & Tarrant, 2011). Many weeds are in this category (e.g., Scotch Broom, *Cytisus scoparius*, Parker, 1997; Simpson, Gross, & Silberbauer, 2005). The increases in plant movement around the world, often facilitated by humans, provide an opportunity to study the reproductive requirements of pioneer species and their interactions with native and naturalized biota in de novae populations. Some of these plant species will become invasive, and in many cases, alien pollinators facilitate population expansion (e.g., *Phyla canescens*, Gross, Gorrell, Macdonald, & Fatemi, 2010). The opportunity to harness the knowledge of pollination requirements into post‐invasion management strategies has so far been ignored and is at risk of further neglect with the global decline in some domesticated pollinators (e.g., *Apis mellifera*) affording them special status, even though they cause damage in some locations (Paini, 2004; Shavit, Dafni, & Ne'eman, 2009). We also contend that the potential impacts of pollination services involving biocontrol agents have been overlooked, adding to a large gap in our understanding of the consequences that range‐shifting insects, including pollinators, have in novel ecosystems.

Zoophilous invasive flora often have flowers adapted for generalist pollination and are therefore attractive to a wide range of pollinators which may, in turn, visit the flowers of numerous other species (Morales & Traveset, 2009). Consequently, invasive floras are often assimilated into native pollination networks, potentially becoming important or embedded species once established within an ecosystem (Memmott & Waser, 2002). This assimilation of exotic flora can exert both positive and negative effects on native plant pollination because competition for pollinators may reduce native floral fecundity through pollinator limitation and heterospecific pollen transfer (Chittka & Schürkens, 2001; Fang & Huang, 2013; Ghazoul, 2004; Traveset & Richardson, 2006; Waser, 1978). However, facilitation may also occur where exotics attract or sustain diverse or abundant pollinator assemblages (Bartomeus, Vilà, & Santamaría, 2008; Chung, Burkle, & Knight, 2014).

*Chrysanthemoides monilifera* ssp. *rotundata* (DC.) Norl., or Bitou Bush, is one of 32 species recognized as a *Weed of National Significance* in Australia (Thorp & Lynch, 2000). Native to the east coast of South Africa, the exact date that Bitou Bush colonized eastern Australia is not known, but it may have been cultivated in Sydney by 1852 (Gray, 1976). The first herbarium record associated with its spread in NSW is from 1908 (*Mayor of Stockton*,* s.n*. 1908, NSW133399, *NSW*), and it was gazetted as a noxious plant by 1909 (in the Stockton area, central coast, NSW, Lee, 1909). It was later promoted as a useful species for the restoration of sand dunes in that region (Mort & Hewitt, 1953; Sless, 1958; Weiss, Adair, Edwards, Winkler, & Downey, 2008). In 2001, it was estimated that 80% of the NSW coastline was invaded by Bitou Bush (Thomas & Leys, 2002). A Threat Abatement Plan for Bitou Bush was released in 2006 (DEC, 2006) which identified over 150 native plant species threatened by Bitou Bush and Bone seed (*Chrysanthemoides monilifera* ssp. *monilifera*) in NSW. As outlined by Downey, Holtkamp, Ireson, Kwong, and Swirepik (2007), a biocontrol program gained momentum from 1984. A series of surveys conducted in South Africa between 1987 and 1990, identified 17 phytophagous insects and two pathogens as potentially suitable biocontrol agents of *C. monilifera* in Australia (Downey et al., 2007 and see Scott, 1996). Of these, six have been released, with four establishing successfully. These include the Bitou Tip Moth (*Comostolopsis germana*), Bitou Tortoise Beetle (*Cassida* sp.), Bitou Seedfly (*Mesoclanis polana*), and Bitou Leaf Roller Moth (*Tortrix* sp.; Downey et al., 2007).

Despite numerous studies identifying the causes, consequences, and potential management strategies relating to Bitou Bush invasions, very few studies have examined specific life‐history traits and their impact on the control and management of the species, or provided scientific evaluation of the control methods currently employed (Lindenmayer et al., 2015). Important work has been conducted on post‐pollination events. For example, previous investigations have found that while adventitious budding of prostrate stems allows Bitou Bush to reproduce vegetatively (Weiss et al., 2008), the primary mode of dispersal and postdisturbance regeneration occurs via seed (Weiss, 1984), with adult plants capable of producing 3,545 ± 600 viable seeds/m^2^ annually (Weiss, 1984). Meanwhile, a broad range of native and exotic vertebrate fauna have been recorded as fruit dispersers (Gosper, 1999; Meek, 1998). Consequently, limiting seed production has been identified as an important means of controlling and preventing further range expansion of the species (Noble & Weiss, 1989) and thus served as justification for the introduction of the seed predator *Mesoclanis polana* in 1996 for biocontrol purposes (Stuart, Kriticos, & Ash, 2002). Yet despite this, knowledge relating to the fertilization processes in *C. monilifera* ssp. *rotundata*, including the breeding system, pollinator dependence and efficacy, remains at present, highly anecdotal. Thus, our objectives were to determine (1) whether or not *Chrysanthemoides monilifera* ssp. *rotundata* requires pollinators to set seed (and to what degree the capacity for fruit production varies among populations when fruit production is compared with glasshouse conditions); (2) the identity, abundance and diversity of pollinators in the field; and (3) whether or not pollinators would be available in areas outside of the current range of Bitou Bush (range extension areas). The latter is particularly important with current Bitou Bush populations on the coast being in close proximity to several vulnerable ecosystems (Laurance et al., 2011) and climatic modeling revealing that Bitou Bush could establish in new regions under scenarios of climate change (Beaumont, Gallagher, Leishman, Hughes, & Downey, 2014). We hypothesized that Bitou Bush would have a facultative breeding system (an optional requirement for pollinators to set seed), and flowers adapted for generalist pollination. It would therefore interact directly with a wide range of pollinators and thus indirectly with native flora within its immediate environment. Any information about the realized breeding system and impacts on native pollination networks may therefore serve to better inform management and control strategies in the future.

## 2 METHODS

### 2.1 Study species

*Chrysanthemoides monilifera* ssp. *rotundata* (DC.) Norl. (Bitou Bush) is an erect, shallow‐rooted, densely branched perennial shrub to 2 m in height. Monoecious inflorescences (flower‐heads) of yellow florets are produced year round (peak August–December) in terminal corymbs. From our study populations, we determined that a flower‐head consists of 5–20 female ray florets (mode = 13), with a bifurcate stigma and ligules 13–19 mm in length; with 30–72 male (pseudohermaphrodite with abortive ovaries) inner disk florets that open sequentially from the outer to the center of the head (centripetal maturation). The female ray florets open on day 1, after ligules have unfurled, and can remain receptive until after anthesis of all male florets. Styles elongate with flower‐head age. Anthesis in male florets occurs sequentially from outer to inner florets from day 1 to day 8 with a peak of new florets on day 2 (Fig. S1). Flower‐head begin to wilt between 6 and 8 days after anthesis. Flower‐heads strongly absorb ultraviolet light (Fig. S2), suggesting that insects are likely to be involved in pollination. Male florets have very small amounts of nectar 0.055 ± 0.005 μl (pooled from 10 florets) at a 59.72 ± 0.61% sucrose equivalent concentration (Table S1 *N* = 4 populations, 74–160 samples each pooled from 10 florets) and female florets have no measurable nectar (Table S1), suggesting that pollinators could be provisioned by male flowers but may spend less time at female florets. Each ray floret may produce a single purplish‐black succulent globose–ellipsoid fruit, 6–8 mm in diameter. Within each fruit, a single hard, bone‐colored seed is produced. The species is capable of vegetative reproduction, which occurs via layering (Weiss et al., 2008).

### 2.2 Does Bitou Bush need pollinators?—breeding system, variability in floral characters, and fruit set

We took cuttings from *Chrysanthemoides monilifera* ssp. *rotundata* plants in 2015 and 2016 from six populations found over a 165 km range on the eastern Australian coastline. The populations were located among sandy dunes or rocky outcrops from the Arrawarra Headland (30°03′ 32.02″S, 153°12′ 10.99″E, *n* = 5 plants), Woolgoolga Headland (30°07′ 11.36″S, 153°12′ 18.48″E, *n* = 10), Hungry Head beach (30°32′ 51.96″S, 153°01′ 37.29″E, *n* = 10), Bongil Bongil beach (30°22′ 53.54″S, 153°05′ 36.77″E, *n* = 9), Tucker's Rock road (30°28′ 38.75″S, 153°02′ 22.87″ E, *n* = 8) to Port Macquarie beach (31°27′ 33.74″S, 152°56′ 00.07″E, *n* = 14). Cuttings were taken from plants collected 20 m apart where possible, to minimize sampling the same genet. In the glasshouse at the University of New England (UNE), Armidale, NSW, cuttings were individually labeled and put in a heated propagation bed to stimulate root formation. After root strike, we transferred the plants into 20‐cm‐wide pots (25 cm deep) containing a commercial potting‐mix. Plants were then placed in a sunny glasshouse room (c. 25°C), kept insect‐free with insect screening on the door and with rare errant insects caught by sticky traps hanging in the room. Plants were watered daily, and flowering occurred within a few weeks. We conducted the following pollination treatments: Autogamy (A), where flower‐heads were left unmanipulated to test for automatic selfing; Selfing (S), where self‐pollen was applied from male to female florets in the same head; Geitonogamy (G), where pollen was transferred from male florets on one head to female florets on a different head on the same plant; Outcrossing (X), where pollen was transferred from one head to another on a separate genetic individual (not a clone). All treatments were applied to all plants (see Table 1 for samples sizes). For outcross pollen donors, we used single donors from within the source population or single donors from another population. We noted how many whorls of male florets were open (1–5) at the time of pollination to determine whether flower‐heads were dichogamous (pistil and stamens maturing at different times). Hand‐pollination results were recorded as ratios of fruit set to female florets treated, and for among population comparisons, dried fruits were weighed as a measure of pollination success and fruit quality using a Sartorius MSE3.6P‐000‐DM Cubis Micro Balance.

#### 2.2.1 Fruit set in coastal naturalized populations

We scored natural fruit set in three populations (Hungry Head Sand Dunes (HHSD), Hungry Head Cabins (HHC), and sand dunes near Tucker's Rock (TRSD)) in November 2016. We surveyed female flower number and fruit‐to‐flower ratios on flower‐heads available for open pollination (HHSD *n* = 145 flower‐heads, *N* = 29 plants; HHC *n* = 52, *N* = 40; TRSD *n* = 102, *N* = 8). The HHSD and HHC populations are separated by a road, a lagoon, and a woodland and are 0.60 km apart, and these populations are c. 19 km from the TRSD population.

#### 2.2.2 Fruit set in a range extension, inland population

To test whether pollinators are available in a range extension area, we also scored fruit‐to‐flower ratios on potted plants from our Arrawarra population that we left in the open at Armidale, NSW (30⁰29′12.87″S; 151⁰38′13.78″E), a location where Bitou Bush has not naturalized and c. 130 km from the nearest coastal population. We grouped together clones of nine plants on outdoor tables and watered them daily. We measured fruit‐to‐flower ratios over a 6 week period (30 November 2016–7 January 2017, *n* = 127 flower‐heads, *N* = 9 plants). As controls, we bagged 34 immature flower‐heads (just before petal expansion) over the duration of the experiment to check for autogamous fruit set.

### 2.3 What visits the flowers of—*Chrysanthemoides monilifera* ssp. *rotundata* and are they pollinators?

#### 2.3.1 Floral visitors in naturalized coastal populations

We used camcorders (Sony Handycam models HDR‐XR160, HDR‐PJ540, and FDR‐AXP35 4K, Gross et al., 2010) to record floral visitations to *Chrysanthemoides monilifera* ssp. *rotundata* over the 2015/2016 flowering seasons at Arrawarra Headland, Woolgoolga, Tucker's Rock and Hungry Head. From these videos, we gathered the following data upon review of the footage; the identity of floral visitors, total time spent visiting a head, resource collected and behavior when at the flowers.

Floral visitors were classified into groups using the terminology developed to characterize floral larceny (Inouye, 1980; Irwin, Bronstein, Manson, & Richardson, 2010). According to the frequency of visits and behavior, we included potential pollinators (PP), nonpollinators (NP), thieves (Th), or predators (Pr). This approach is pragmatic for revealing pollinators in a system (Jacobs et al., 2010) but requires more detailed testing to rank effectiveness (Gross & Mackay, 1998 and see below). Visitors classified as potential pollinators (PP) were those that made contact with both male (anthers) and female (stigmas) reproductive structures, with pollen on the body demonstrating the ability to transport pollen within and between flowers of separate plants. The latter is recognized as an important step in discerning pollinators from nonpollinators (Popic, Wardle, & Davila, 2013). Nonpollinators (NP) were visitors that did not contact floral reproductive structures and were not observed to collect pollen and/or nectar. Thieves (Th) were visitors observed collecting pollen and/or nectar without making reliable contact with both male and female reproductive structures. Predators (Pr) were incidental visitors observed attempting to predate or parasitize other floral visitors, which may or may not have contacted floral reproductive structures in the process. In some floral systems, pollinators may not contact the stigma during every foraging bout (Gross & Kukuk, 2000) but we did not observe this behavior with those insects visiting Bitou Bush flower‐heads that we classified as PP.

Insects were collected with an entomological net and/or bottle and then euthanized with ethyl acetate. Insects were then mounted in pinning boxes or kept in 70% alcohol and identified using reference collections at the University of New England (UNE), field guides (e.g., Braby, 2004; Zborowski & Storey, 2010), on line resources (e.g., www.ala.org.au/, www.padil.gov.au/; www.bowerbird.org.au/) or expert knowledge.

#### 2.3.2 Pollen loads

For the four most common floral visitors, in March–April 2017 at Hungry Head, we determined their potential for transferring pollen by counting a subsample of the pollen grains carried by them. We collected foraging insects (*N* = 1–13 individuals per species) on flower‐heads, and we removed pollen from their bodies by swabbing them through a droplet of pollen dye (5% solution of lactophenol aniline blue [5 ml 5% aqueous aniline blue (aniline Blue W.S. (CI 42775, Difco 3024, Detroit, MI), 20 ml phenol, 20 ml lactic acid, 40 ml glycerin, and 20 ml water]) placed on a microscope slide. After swabbing, a cover slip was applied to the droplet and then sealed with clear nail polish and the slide labeled. For the most common visitor (Bitou Seedfly, see below), we applied an extra step to investigate whether foragers deposit pollen onto stigmas as visitation rates may not equate with pollinator effectiveness (King, Ballantyne, & Willmer, 2013 but see Gross & Mackay, 1998). A freshly opened flower‐head (*N* = 11) that was in early female phase (to minimize self‐pollen from any open male flowers confounding pollen counts) was picked and held next to a flower‐head where a Bitou Seedfly was foraging. The close proximity of flower‐heads facilitated the animal moving from the first flower‐head to the experimental flower‐head, where we allowed it to forage freely. After the fly flew away, we excised styles from the flower‐head and traced them through a droplet of pollen dye (as above) before sealing with a cover slip. Controls were freshly picked flower‐heads, where we did not introduce floral visitors to them (*N* = 15 flower‐heads) but where styles were treated as above. We viewed the prepared slides of experimental and control treatments with a compound microscope (Leica DME microscope, ×40 magnification) and aimed for a full pollen count by moving the stage back and forth across each slide in nonoverlapping “transects,” while simultaneously sweeping the field of view and tallying the number of pollen grains in view.

#### 2.3.3 Floral visitors in a range extension, inland population

We used camcorders (see above) on 4 days (5–6 December 2016, 22 December 2016, 7 January 2017) for a total of 23.5 hr to record floral traffic on our plants placed outside the glasshouses of UNE, Armidale. We also opportunistically took digital images and insect samples of floral visitors to the flower‐heads for identification.

### 2.4 Data analyses

A one‐way analysis of variance (ANOVA) was computed to compare fruit production against the number of whorls of male flowers (a surrogate for flower‐head age, see above). We used ANOVA to investigate plasticity in floral characters (petal number, fruit‐to‐flower ratios, fruit mass) using glasshouse populations to provide a baseline for interpreting field experiments. To investigate differences in pollen loads carried by floral visitors, we used ANOVA. We used ANOVA to compare foraging behavior (time spent at flowers) and abundance for those floral visitors found in both naturalized, coastal populations and the range extension population at Armidale. For the ANOVAs, where necessary, some variables were log‐transformed to improve normality and homoscedasticity (fruit mass was log‐transformed and visitation data 1/SQRT transformed). Data were analyzed with the statistics program *Statgraphics Plus* version 3.5^®^.

## 3 RESULTS

### 3.1 Breeding system, variability in floral characters, and fruit set

**Figure 1:**
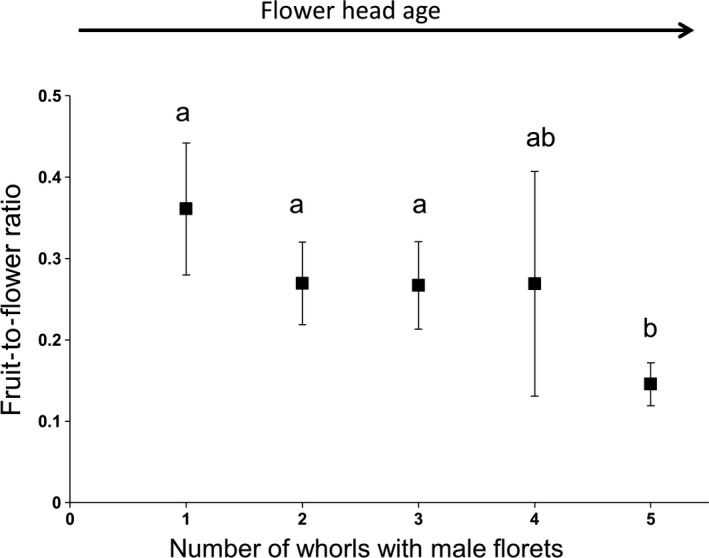
Fruit‐to‐flower ratios for 181 outcrossing events undertaken in the glasshouse at UNE between March 2016 and March 2017, where female flowers were of different ages. Flower age was scored against the number of open whorls of male florets, with category 1 a fresh flower‐head with 1 whorl of male florets and category 5 an older flower‐head with 5 whorls of male florets. Data points with different letters were significantly different at *p* < .01 using post hoc LSD (Least Significant Difference).

Bitou Bush only formed seed with outcross pollen (Table 1). This result was consistent across six populations. Within the outcross pollination treatment, glasshouse fruit set was mostly at least 45% across populations with the highest level recorded with the Bongil Bongil provenance at 63.63%. Although all outcrossed flowers were treated with pollen, conversion to fruits at the head level ranged from 26.68% to 65.69%. With pooled data across populations, flower‐heads were found to be protogynous, and female flowers were more likely to set seed at the early stages of anthesis (*F*_4, 181_ = 3.03, *p* = .02), when only the first 1–3 whorls of male flowers were open (Figure 1).

**Table 1:** Glasshouse breeding system results for *Chrysanthemoides monilifera* ssp. *rotundata*. Hand‐pollination experiments were conducted on plants from six populations between March 2016 and March 2017. Fructification (%) is the percentage of flower‐heads that produced at least one fruit. Seed set (%) is measured as the proportion of seeds developing per flower on an infructescence that had at least one fruit. *N*,* n* are sample sizes. Fruit mass is in milligrams (mg)

Population Treatment	Plants (*N*)	Flower‐heads (*n*)	Frutification (%)	Seed set (%)	Mean fruit mass ± 1 *SE* (mg) (*n*)
Arrawarra					
Autogamy	5	58	0	0	—
Selfing	3	22	0	0	—
Geitonogamy	3	13	0	0	—
Outcross	5	39	48.71	65.69	44.03 ± 1.00 (106)
Bongil Bongil					
Autogamy	5	16	0	0	—
Selfing	3	16	0	0	—
Geitonogamy	3	22	0	0	—
Outcross	5	33	63.63	44.71	55.52 ± 2.12 (95)
Hungry Head					
Autogamy	4	15	0	0	—
Selfing	4	10	0	0	—
Geitonogamy	4	10	0	0	—
Outcross	4	15	46.67	28.47	55.01 ± 2.62 (10)
Port Macquarie					
Autogamy	11	30	0	0	—
Selfing	3	11	0	0	—
Geitonogamy	6	10	0	0	—
Outcross	10	38	23.68	26.68	48.98 ± 2.63 (26)
Tucker's Rock					
Autogamy	6	32	0	0	—
Selfing	7	18	0	0	—
Geitonogamy	5	26	0	0	—
Outcross	7	52	46.15	41.89	48.98 ± 1.66 (140)
Woolgoolga					
Autogamy	8	51	0	0	—
Selfing	6	37	0	0	—
Geitonogamy	7	35	0	0	—
Outcross	8	90	45.56	40.08	51.71 ± 1.21 (212)

When grown in the uniform conditions of the glasshouse, the number of female flowers on a head was a plastic character that varied significantly among populations (Fig. S3, *F*_5, 191_ = 8.68, *p* < .001). When comparing identical plants growing inside and outside the glasshouse, the Arrawarra provenance produced significantly different numbers of female flowers depending on location (inside or outside) and a significant interaction of factors indicates the plasticity of female flower number at the plant level (plant × location, *F*_4, 160_ = 3.25, *p* < .01).

Fruit‐to‐flower ratios in the four open pollinated situations (HHC, HHSD, TRSD and the Arrawarra plants outside the glasshouse) ranged from 12% to 44% and varied significantly due to the very low fructification at TRSD (12%, Fig. S3, *F*_3, 422_ = 44.56, *p* < .001). Fruit set in the glasshouse, where flowers received only one application of pollen, was significantly lower than in the open (coastal and Armidale) field situations where individual flower‐heads were open to pollination events over many days (pooled glasshouse 22.81 ± 2.25, *n* = 186 flower‐heads, pooled open 33.04 ± 1.22, *N* = 426, *F*_1, 610_ = 18.45, *p* < .001).

Fruits from the outcrossed treatments did not vary significantly in their mass among the populations in which we only used intrapopulation pollen donors (four populations, *F*_3, 279_ = 0.34, *p* = .79, pooled fruit mass 48.35 ± 0.91 mg, *N* = 283). For populations in which we used both intra‐ and interpopulation pollen donors (four populations), we found a significant interaction between population and pollen source (intra vs inter, interaction *F*_3, 453_ = 6.14, *p* = .0004), due to Arrawarra being the only population with lower fruit mass from interpopulation outcrosses (Table S2).

### 3.2 Floral visitors—*Chrysanthemoides monilifera* ssp. *rotundata*

#### 3.2.1 Naturalized coastal populations

**Figure 2:**
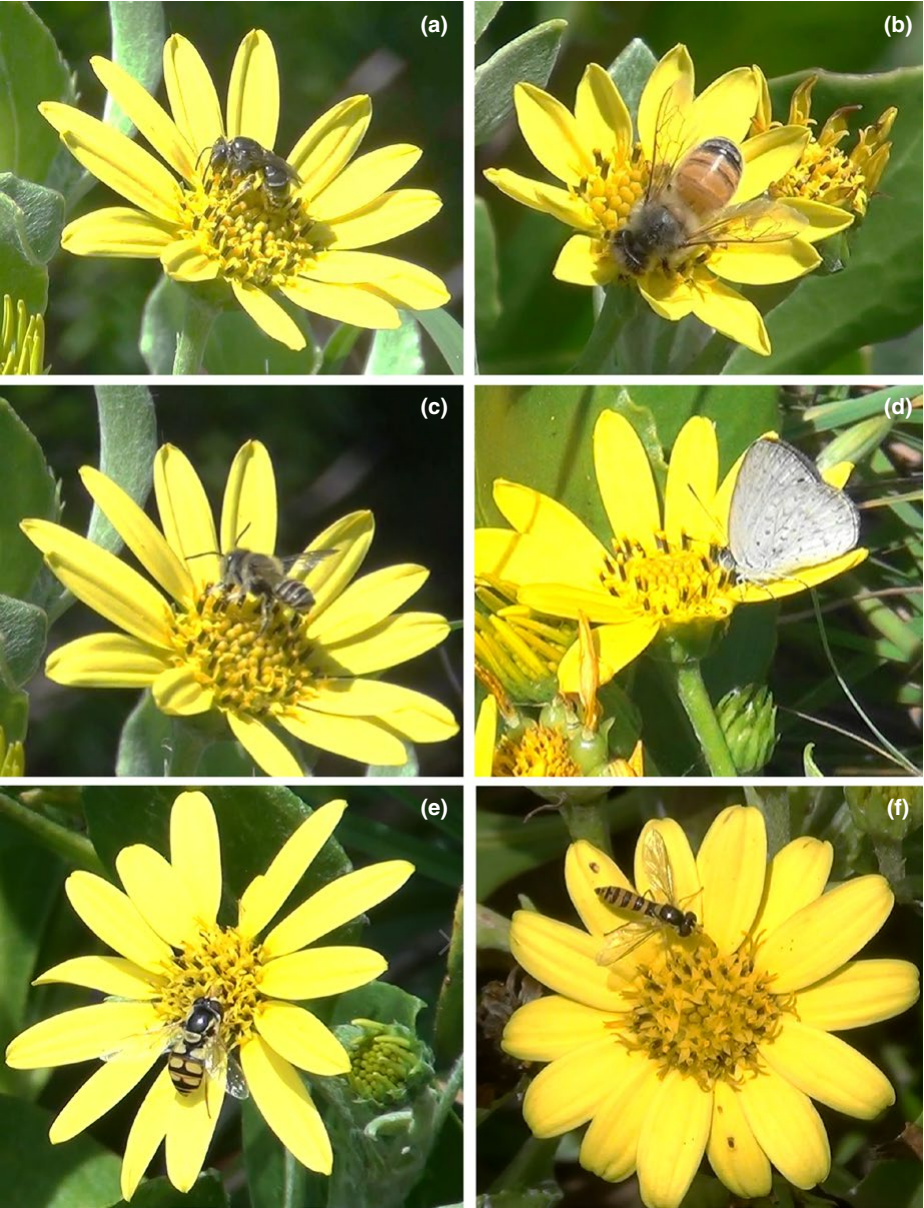
Pollinators of *Chrysanthemoides monilifera* ssp. *rotundata* (Bitou Bush): (a) *Lipotriches flavoviridis* collecting nectar and pollen, (b) Honeybee *Apis mellifera* collecting nectar and pollen, (c) *Megachile simplex* (d) *Zizina otis labradus* collecting nectar (e), *Simosyrphus grandicornis* (f), *Sphaerophoria macrogaster*

**Figure 3:**
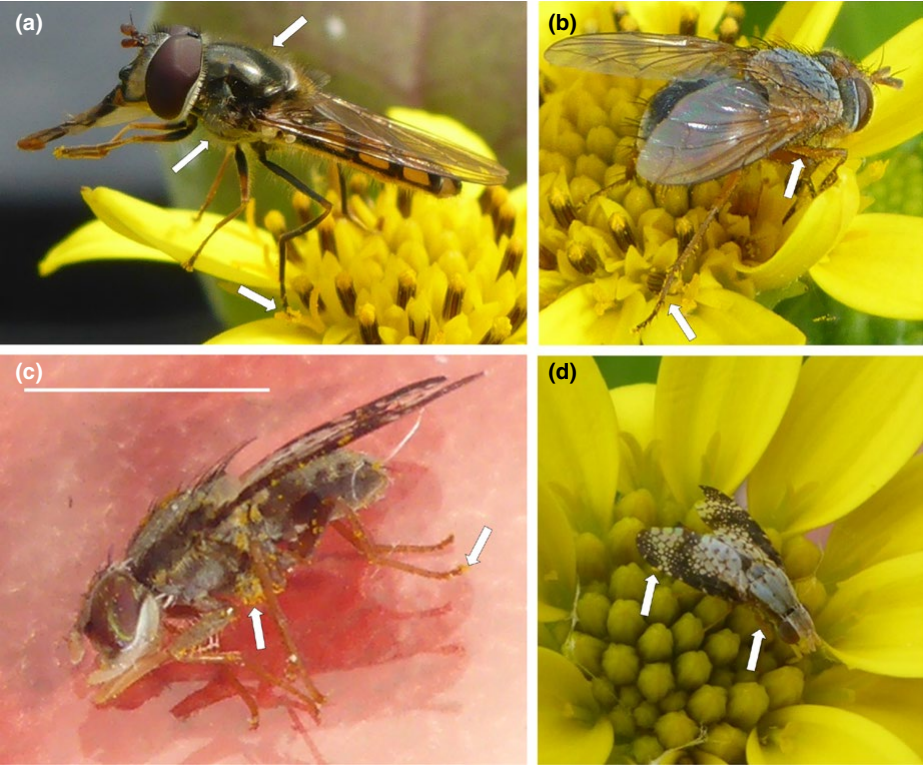
Bitou Bush pollen on the bodies of three species of fly (as indicated by arrows); (a) *Melangyna viridiceps*, note the tarsus laden with pollen touching the stigma and (b) *Calliphora augur*, note the pollen laden tarsus touching an anther and close to a stigma and (c) *Mesoclanis polana* (Bitou Seedfly) positioned on a glass plate to show the pollen on the ventral side of the body, scale bar is 2.5 mm and (d) foraging at a flower‐head

**Figure 4:**
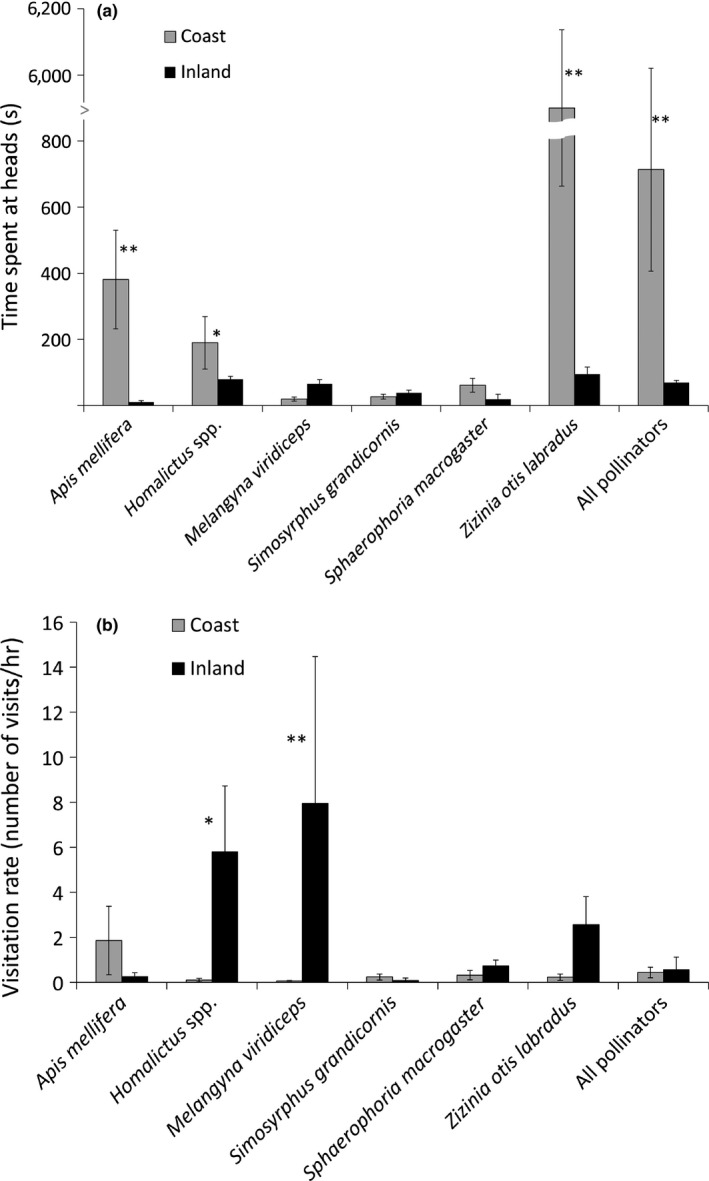
(a, b) A comparison of the behavior of six pollinators (*Apis mellifera*,* Homalictus* spp., *Melangyna viridiceps*,* Simosyrphus grandicornis*,* Sphaerophoria macrogaster*,* Zizinia otis labradus*) when visiting *Chrysanthemoides monilifera* ssp. *rotundata* in two populations. We used a naturalized, coastal population, and an inland population at Armidale that was artificially established and represents a hypothetical range extension for Bitou Bush. The populations are separated by c. 130 km. (a) Time spent at flower flower‐heads (mean time (s) ± 1 *SE*) by pollinators and all pollinators combined and (b) their visitation rates to flower‐heads (mean number of visits per hour ± 1 *SE*). Samples sizes *N* = 150 hr in the naturalized population on the coast and *N* = 23.5 hr in the artificial population inland. Paired *t* test results with significant results for different pollinator types are indicated by *p < .05, **p < .001

In total, we collected 150 hr of floral visitation data over the two seasons (October–December 2015 and November 2016) from 41 plants. We recorded 557 visitors from 35 species of arthropods visiting the flower‐heads of Bitou Bush (Table 2). Visitors included Hymenoptera (bees, wasps, and ants ~13 spp.), Diptera (8 spp.), Lepidoptera (5 spp.), Coleoptera (2 sp.), Hemiptera (2 sp.), Thysanoptera (~3 spp.), and Araneae (spiders) (~2 spp.) (Table 2, Figures 2, 3, 4). Within the Hymenoptera, honeybees (*Apis mellifera*) were the most frequent floral visitor (Table 2, Figure 4b), acting as a potential pollinator during visits by contacting stigmas and anthers, and visiting flower‐heads on different individuals. Native bees, such as large bees of the genus *Xylocopa* and small bees such as *Lipotriches flavoviridis* (Table 2, Figure 4a), also visited Bitou Bush. Ants spent the most time of all visitors on flower‐heads (Table 2), but their behavior was not as a potential pollinator because they only walked between the flower‐heads or sheltered under the flowers and they often chased away potential pollinators.

**Table 2:** Floral visitors, the number of visits (*N*), visitation rates (number of visitors per hour), and visitation time at flower‐heads (mean seconds spent at flower‐heads ± 1 *SE*) to *Chrysanthemoides monilifera* ssp. *rotundata* in 150 hr of pooled observations from the coast (Arrawarra Headland, Woolgoolga, and Hungry Head) and in 23.5 hr of pooled observations from the artificial glasshouse population set up outside the glasshouses at UNE, the inland population at Armidale, NSW. Behavior: NPP = Not a Potential Pollinator, PP = Potential Pollinator, Pre = Predator of another floral visitor, Th = Thief, NA = undetermined. The most common pollinator (number of visitors per hour) at each location is bolded

Order	Family	Taxon	*N*	Location	Visitation rate (visitors/hr)	Visitation time (s)	Behavior
Araneae	Salticidae	*Simaetha* sp.	2	Coast	0.01	1296.50 ± 404.50	Pre
	Thomisidae	*Thomisus* spp.	7	Coast	0.05	546.00 ± 399.13	Pre
Coleoptera	Coccinellidae	*Coelophora inaequalis*	1	Coast	0.01	5	Th
	Curculionidae	*Meriphus* sp.	5	Coast	0.03	2592.60 ± 2444.60	Th
Diptera	Bombyliidae	*Geron* sp.	1	Coast	0.01	420	PP
		*Villa* sp.	2	UNE	0.09	34.00 ± 13.00	PP
	Calliphoridae	*Calliphora augur*	2	UNE	0.09	12.50 ± 10.50	PP
		*Calliphora centralis*	31	UNE	1.32	28.61 ± 4.48	PP
		*Chrysomya megacephala*	7	UNE	0.3	53.43 ± 30.95	PP
		*Chrysomya* sp.	1	Coast	0.01	14	Th
	Lauxaniidae	*Homoneura* sp.	12	Coast	0.08	99.33 ± 40.00	NPP
		*Sapronyza* sp.	1	Coast	0.01	59	NPP
	Muscidae	*Hydrotaea* sp.	8	UNE	0.34	53.75 ± 27.09	PP
		*Hydrotaea* sp.	1	Coast	0.01	1	NPP
		*Musca vetustissima*	3	UNE	0.13	11.33 ± 3.93	PP
	Syrphidae	*Eristalis tenax*	4	UNE	0.17	27.00 ± 15.80	PP
		*Melangyna viridiceps*	5	Coast	0.03	19.60 ± 6.28	PP
		*Melangyna viridiceps*	61	UNE	2.6	66.07 ± 12.52	PP
		*Simosyrphus grandicornis*	24	Coast	0.16	27.00 ± 7.31	PP
		*Simosyrphus grandicornis*	20	UNE	0.85	38.55 ± 8.81	PP
		*Sphaerophoria macrogaster*	15	Coast	0.1	61.53 ± 21.08	PP
		*Sphaerophoria macrogaster*	4	UNE	0.17	19.50 ± 14.86	PP
	***Tephritidae***	***Mesoclanis polana***	***138***	***Coast***	***0.92***	***443.91 ± 391.4***	***PP***
Hemiptera	Lygaeidae	*Crompus* sp.	136	Coast	0.91	199.58 ± 20.08	Th
		*Crompus* sp.	11	UNE	0.47	658.18 ± 151.32	Th
	Miridae	*Taylorilygus* sp. 1	7	Coast	0.05	2657.86 ± 2138.71	Th
Hymenoptera	Apidae	*Amegilla* sp.	1	Coast	0.01	1	PP
		*Apis mellifera*	17	Coast	0.11	381.35 ± 148.87	PP
		*Apis mellifera*	7	UNE	0.3	10.71 ± 4.29	PP
		*Exoneura sp*.	1	UNE	0.04	27	PP
	Braconidae	Microgastrinae spp.	6	Coast	0.04	104.33 ± 77.63	PP
		Braconidae sp.	1	Coast	0.01	60	PP
	Formicidae	*Iridomyrmex* sp.	15	UNE	0.64	30.53 ± 4.77	Pre
		*Iridomyrmex* spp.	77	Coast	0.51	105.16 ± 52.86	NPP
	***Halictidae***	***Homalictus sphecodoides***	***104***	***UNE***	***4.43***	***79.96 ± 8.97***	***PP***
		*Homalictus* spp.	5	Coast	0.03	30.20 ± 18.40	PP
		*Lipotricus flavoviridis*	5	Coast	0.03	1638.20 ± 871.02	PP
	Ichneumonidae	Campopleginae sp.	2	Coast	0.01	1.50 ± 0.50	PP
		Campopleginae sp.	2	UNE	0.09	17.00 ± 5.00	PP
		*Diplazon lactatorius*	4	Coast	0.03	468.50 ± 443.86	PP
	Megachilidae	*Megachile simplex*	7	Coast	0.05	784.00 ± 666.97	PP
	Tiphidae	*Anthobosca* sp.	1	Coast	0.01	1	NA
	Xylocopinae	*Braunsapis* sp.	4	Coast	0.03	850.75 ± 549.42	PP
		*Xylocopa (Koptortosoma)* spp.	8	Coast	0.05	930.50 ± 595.69	PP
		*Xylocopa (Lestis) bombylans*	2	Coast	0.01	2	PP
Lepidoptera	Lycaenidae	*Candalides erinus*	2	Coast	0.01	1	PP
		*Zizinia otis labradus*	10	Coast	0.07	5193.20 ± 2368.41	PP
		*Zizinia otis labradus*	17	UNE	0.72	94.76 ± 21.17	PP
	Nymphalidae	*Vanessa kershawi*	3	UNE	0.13	25.67 ± 10.09	PP
	Pieridae	*Eurema smilax*	5	Coast	0.03	69.20 ± 27.21	PP
		*Pieris rapae*	1	Coast	0.01	11	PP
	Zygaenidae	*Pollanisus subdulosa*	4	Coast	0.03	5946.25 ± 4488.91	Th
Thysanoptera		Thysanoptera spp.	39	Coast	0.26	3615.00 ± 813.43	Th

Within the Diptera, *Mesoclanis polana* (Bitou Seedfly) was the most frequent pollinator observed across all orders and was responsible for 52% of potential pollinator visits to flowers (Table 2, Figure 3c,d) and it was near tenfold more common than honeybees. Bitou Seedfly probed female and male flowers when walking over flower‐heads, they contacted stigmas and anthers, and pollen was observed on their bodies (Figure 3c,d). They moved between flower‐heads and other plants. Significantly more Bitou Seedflies were detected at Hungry Head than at other sites (Hungry Head *N* = 138, other sites pooled *N* = 12). Other flies also acted as potential pollinators, including the hoverflies *Melangyna viridiceps, Simosyrphus grandicornis*, and *Sphaerophoria macrogaster*. Other species within Diptera were of small size and often did not contact the reproductive structures of the flowers, but often robbed floral resources (Table 2).

In the Lepidoptera, most of the visitors behaved as pollinators, except for 1 moth, *Pollanisus subdulosa*, which despite the high time spent at flowers did not collect pollen but consumed nectar (Table 2)**.** In the remaining orders sampled, notwithstanding the high total time of visits, they did not act as potential pollinators, often only consuming pollen or floral structures in the flower‐heads (Thysanoptera spp.; Hemiptera; other small insects), or in the case of Araneae, the flower‐heads were used as a platform to seize floral visitors for prey, disrupting potential pollinators (Table 2).

In summary, of the 35 species of arthropods that visited flower‐heads, 21 species of insect collected pollen and probed the female flowers of Bitou Bush. At the coast, the most frequent visitor was the introduced fly, *Mesoclanis polana* (Bitou Seedfly, 52% of visits), followed by a native hoverfly *Simosyrphus grandicornis* (9%) and the introduced European Honeybee *Apis mellifera* (6.5%).

#### 3.2.2 Pollen loads

Body swabs from the most frequent floral visitors at Hungry Head showed that all individuals carried Bitou Bush pollen (Table 3). The introduced honeybee, *Apis mellifera,* carried the most pollen (Table 3). For the most frequent floral visitor, Bitou Seedfly, we found incontrovertible evidence that individual flies are pollinators (Figure 3c,d). They deposited significant (*F*_1, 21_ = 15.48, *p* = .0008) amounts of pollen onto the stigmas of freshly opened flowers when compared with background counts from stigmas excised from freshly opened control flowers (mean pollen grains ± 1 *SE*, fly flowers 152.36 ± 34.77, *N* = 11, control flowers 13.33 ± 5.29, *N* = 15). We also detected other plant species in the pollen samples from Bitou Seedfly (data not shown, <5% of pollen grains counted) including *Banksia integrifolia* and myrtaceous pollen.

**Table 3:** *Chrysanthemoides monilifera* ssp. *rotundata* pollen (mean number of pollen grains ± 1 *SE*, minimum, maximum) swabbed from the bodies of floral foragers at Hungry Head in March–April 2017. *N* = number of insects sampled

Order	Floral Visitor	*N*	Mean number of pollen grains ± *SE*	Minimum	Maximum
Diptera	*Melangyna viridiceps*	4	583 ± 205.85	1,302	2,496
	*Mesoclanis polana*	13	248 ± 72.94	9	868
	*Simosyrphus grandicornis*	1	22	22	22
Hymenoptera	*Apis mellifera*	10	2,327 ± 684.61	488	8,172
	*Homalictus* spp.	2	1,221 ± 176	1,045	1,397

#### 3.2.3 Floral visitors in a range extension, inland population

We found that 18 species of insects used the flower‐heads of potted Bitou Bush plants (Table 2) in Armidale. The most frequent visitor was the native bee *Homalictus sphecodoides*, followed by the native hoverfly, *Melangyna viridiceps* (Table 2).

#### 3.2.4 Coastal naturalized population vs artificial inland population

There were six species of pollinators in common between the coast and the inland populations of Bitou Bush (Table 2, Figure 4a,b, *Homalictus* species were pooled). We found that the time spent foraging on flower‐heads varied for some pollinators with location (interaction *F*_5, 290_ = 4.70 *p* < .001, Figure 4a) and was most marked for *Apis mellifera* and *Zizinia otis labradus* which spent more time foraging on flower‐heads on the coast than inland (Figure 4a). Although we had fewer hours of observation inland (inland 23.5 hr vs coastal 150 hr), some pollinators were more abundant on plants flowering inland at Armidale than in the naturalized coastal populations (interaction *F*_5, 40_ = 2.73, *p* = .03, Figure 4b).

## 4 DISCUSSION

The utility of pollinator knowledge for integrated weed‐management strategies has been overlooked. Our work shows that for Bitou Bush (*Chrysanthemoides monilifera* ssp. *rotundata*) this has been a serious oversight. Bitou Bush is a weed of National Significance in Australia (Thorp & Lynch, 2000). We are the first to show that the species needs pollinators to effect seed set and that native and exotic insects, including species introduced for the biocontrol of Bitou Bush, are pollen vectors in northern NSW. Our breeding system findings are in contrast to commentary that pollinators are not essential for “pollination” (i.e., seed set, Weiss et al., 2008) and our results were consistent across six populations. We also recorded that the flower‐heads are protogynous, in contrast to commentary (Weiss et al., 2008), and we found that the older flower‐heads are less likely to set seed compared with younger flower‐heads.

A range of insects are capable of pollinating Bitou Bush in our study populations and this is not surprising as the Asteraceae often have a diverse floral visitor assemblage to their flowers (e.g., table 2 in Hingston & McQuillan, 2000). Significant pollen loads were carried by bees, particularly introduced honeybees. Much of the pollen load on the introduced honeybee was contained in the corbiculae and while these pollen grains may not be available for pollination, such grooming may not lower their effectiveness as pollinators (Davis, 1992). However, the most frequent pollen vector was *Mesoclanis polana* (Bitou Seedfly) introduced to parasitize Bitou Bush seed. The Bitou Seedfly was almost tenfold more common than honeybees at flower‐heads. A single foraging event at a flower‐head by *M. polona* resulted in more than 130 pollen grains being deposited on stigmas, approximately half the pollen that they carry on their bodies. In a ten‐hour day, a flower‐head would receive nearly 10 individuals of *Mesoclanis polana* and the potential deposition of an estimated 1300 pollen grains to stigmas.

We have not found any data or reports on the pollination requirements of *Chrysanthemoides monilifera* ssp. *rotundata* in its native range of the Cape Area in South Africa. Assessments in South Africa for the purposes of biocontrol options in Australia have instead had a focus on ovule predation, post‐pollination events (seed production, seed predation, seed bank densities), population densities and leaf herbivory (Scott, 1996). This has left a gap in our knowledge of the reproductive biology of Bitou Bush. Knowledge of the pollinators in its native range may have revealed the dual role that the Bitou Seedfly can play in the biology of Bitou Bush (adults as pollinators and larvae as seed predators), which in turn may have influenced decisions around the suitability of Bitou Seedfly for introduction into Australia. This omission is unfortunate especially as the Bitou Seedfly is “likely to have little effect on the persistence and recolonization ability of an established Bitou Bush stand.” (Stuart et al., 2002).

We do not know whether *Mesoclanis polana* is an obligate pollinator for Bitou Bush in South Africa, but we postulate that the lack of a specialized floral system implies that these pollinating seed parasites are likely to co‐occur with other pollinators. Sometimes termed as nursery pollination, there are several examples of coevolution between plants and pollinators that are also seed parasites (e.g., obligate systems including figs and fig wasps Janzen, 1979; yuccas and yucca‐moths, Riley, 1892; globe flowers (*Trollius* species, Ranunculaceae), globe flower flies in the genus *Chiastocheta* (Anthomiidae), Pellmyr, 1989; and generalist systems including the Senita cactus (*Lophocereus schottii*) and Senita moths (*Upiga virescens*), Holland & Fleming, 1999 and the Starry campion (*Silene stellata*) an herbaceous perennial, and a Noctuidae moth, *Hadena ectypa*, Kula, Castillo, Dudash, & Fenster, 2014). Seed parasites that are also important pollinators of the same species are thus not unusual. In all of these systems, the seed parasitizing pollinators were usually responsible for more seed production than seed loss, with the average percentage of seeds lost to pollinator offspring ranging between 1% and 60% in published studies of fig/fig‐wasp, yucca/yucca‐moth, senita/senita‐moth and globe flower/globe‐flower‐fly systems (Bronstein, 2001). We therefore suggest that it is important when looking for biocontrol agents that parasitize seeds, that they are checked to make sure that they are not also important pollinators. Further work is required to discern the full floral preferences of Bitou Seedfly as pollen swabs from bodies showed that the species is not faithful to Bitou Bush. Determining whether Bitou Seedfly has also crossed to native species for nursery sites would be important to determine as a possible threat to the native plant species in this coastal ecosystem type, much of which is recognized as under threat. Our work has shown that the behavior of the biocontrol agent should be assessed at all stages of a flower's lifespan.

The evaluation of an invasive plant's breeding system as an a‐priori step in biocontrol programs is not standard practice even though the obligate requirement for pollinators has been demonstrated for several invasive plant species that have detrimental environmental, economic and social impacts (e.g., in Australia *Cytisus scoparius*, Simpson et al., 2005; *Phyla canescens*, Gross et al., 2010). A proposed biocontrol agent for the invasive sister taxon *C. monilifera* ssp. *monilifera*, the Lacy‐winged seed fly (*Mesoclanis magnipalpis*), was released under special “direct release” (Downey et al., 2007). It is concerning that the decision to release the Lacy‐winged seed fly was based on host plants not setting seed in quarantine (and presumably populations of the seed fly could not be raised in quarantine). Plants in captivity that do not set seed may need pollinators—and this should have been a warning about the potential for obligate requirements for pollinators in *Chrysanthemoides monilifera*.

The dogma that weedy species are autonomous of pollinators may have its origins in the misinterpretation of Baker's Rule (Baker, 1967), as recently unpacked by Pannell et al. (2015). Deliberate introductions of a self‐incompatible species alleviates mate limitations that may be inherent in species that have arrived via natural, long‐distance dispersal events (Pannell et al., 2015). In addition, if pollinators that have coevolved with the plant species are added to the mix (this study), or generalists are present (European honeybee, *Apis mellifera* L.) then allogamous invasive species are able to proliferate (see *Phyla canescens*, a successful cosmopolitan species, Gross, Fatemi, Julien, McPherson, & Van Klinken, 2017).

Bitou Bush is pollinated by native and introduced bees, flies and butterflies. It shares some of these bee species with other native species (e.g., sympatric *Hibbertia scandens*, CLGross, unpub data). Our work provides a starting point for a full investigation of pollinator networks in this community.

The impact of changing climates on plant species may mean for some species that a range extension will become possible as the temperature‐limiting effects (e.g., frosts) on plant establishment, growth, and reproduction are ameliorated. Bitou Bush is frost sensitive and would not be able to establish in, for example, the New England area of Australia at present. However, with milder winter temperatures predicted for the area (OEH, 2014), we have shown that should Bitou Bush establish in the area, it would have ready pollinators to effect fruit set. Furthermore, the stochastic plasticity in ray floret numbers and fruit production suggests that the species may be able to rapidly capitalize on new conditions with its ability to be used by widespread and common pollinators.

*Mesoclanis polana* (Bitou Seedfly) was introduced to NSW in 1996 to control seed production in Bitou Bush (Downey et al., 2007) with scant assessment and in disregard to models indicating that satisfactory control could only be achieved if predispersal seed predation could reduce viable seed production by >95% year round (Noble & Weiss, 1989). Seed herbivory by *Mesoclanis polana* has not been detected at this level. It reduces total ovule numbers by c. 31% in native South African populations (table 1 in Edwards & Brown, 1997), and in Australia, the impact is variable and often low (23%–31%, Stuart et al., 2002; 2%–86%, Edwards et al., 2009). The introduction of *Mesoclanis polana* to Australia has not been benign however. Carvalheiro, Buckley, Ventim, Fowler, and Memmott (2008) have shown that species richness and abundance of native seed herbivores were negatively correlated with Bitou Seedfly abundance with an associated increase in the abundance of shared natural enemies (predators & parasitoids). Our results add to this by showing that *M. polana* contributes to ecosystem damage by being a pollinator of one of Australia's Weeds of National Significance. Clearly, the introduction of the Bitou Seedfly has overall been disadvantageous to native ecosystems in Australia.

Alien species have had a global impact on native biodiversity and the economies of natural and agricultural systems, costing world economies an estimated USD$1.4 trillion (Pimentel et al., 2001). The cost of weeds alone to the Australian economy in 2002 were estimated to be as high as AUD$4.5billion (Sinden et al., 2004) and the direct cost of implementing only part of the Bitou Bush and Bone seed NSW Threat Abatement Plan in 2005–2006 was AUD$2,845,500 (DEC, 2006). Biological control agents can be a highly successful and cost‐effective means of controlling pests, as in the successful deployment of *Cactoblastis cactorum* for the control of the extremely destructive weed, *Opuntia stricta*, and other prickly pear species in eastern Australia (Raghu & Walton, 2007). However, the assisted movement of species among continents for biocontrol can often have unforeseen consequences (Ricciardi & Simberloff, 2009; Strong, 1997). The introduction of the cane toad (*Bufo marinus*) to Australia as a biocontrol agent (Easteal, 1981) is often cited as an action that has had and continues to have disastrous consequences for the Australian biota (Shine & Wiens, 2010). Lessons have been learned, but mistakes are still made. Bitou Bush (*Chrysanthemoides monilifera* ssp. *rotundata*) is a species that had inadequate ecological assessment in both the native and naturalized ranges before its widespread use in eastern Australia. Furthermore, its introduced biological control agent, *Mesoclanis polana,* is our latest example of a deliberately introduced species having unexpected, potentially detrimental effects in its non‐native range; in this case increasing the target weed's fitness, as the major pollinator, while having only minor impacts as a seed predator.

## DATA REPOSITORY

The data that support the findings of this study are stored by and available from the University of New England and provided as open access, under a CC‐BY 4.0 license. Data can be accessed from https://doi.org/10.4226/95/599e452ba3c31

## AUTHOR CONTRIBUTIONS

CG conceived the project. All authors contributed to field and laboratory work. All authors contributed to the writing of the article.

## CONFLICT OF INTEREST

None declared.
