# Supplementary material for: Unsuccessful introduced biocontrol agents can act as pollinators of invasive weeds: Bitou Bush (Chrysanthemoides monilifera ssp. rotundata) as an example
Source: Ecol Evol. 2017 Sep 21;7(20):8643–56. doi: 10.1002/ece3.3441 (PMC5648678; doi:10.1002/ece3.3441)
Supplement: Supplementary file 1 [file ECE3-7-8643-s001.docx]

Supplementary files

***Figure S1.*** The timing of anthesis for male florets using 400 flower-heads monitored on plants in the glasshouse at UNE and pooled from five populations (Bongil Bongil, Hungry Head, Port Macquarie, Tucker’s Rock and Woolgoolga). The cumulative number of male florets is the running total of male flowers produced on flower-heads and the number of new male flowers is the number of new male flowers (mean ± 1 SE) open on a flower head each day.


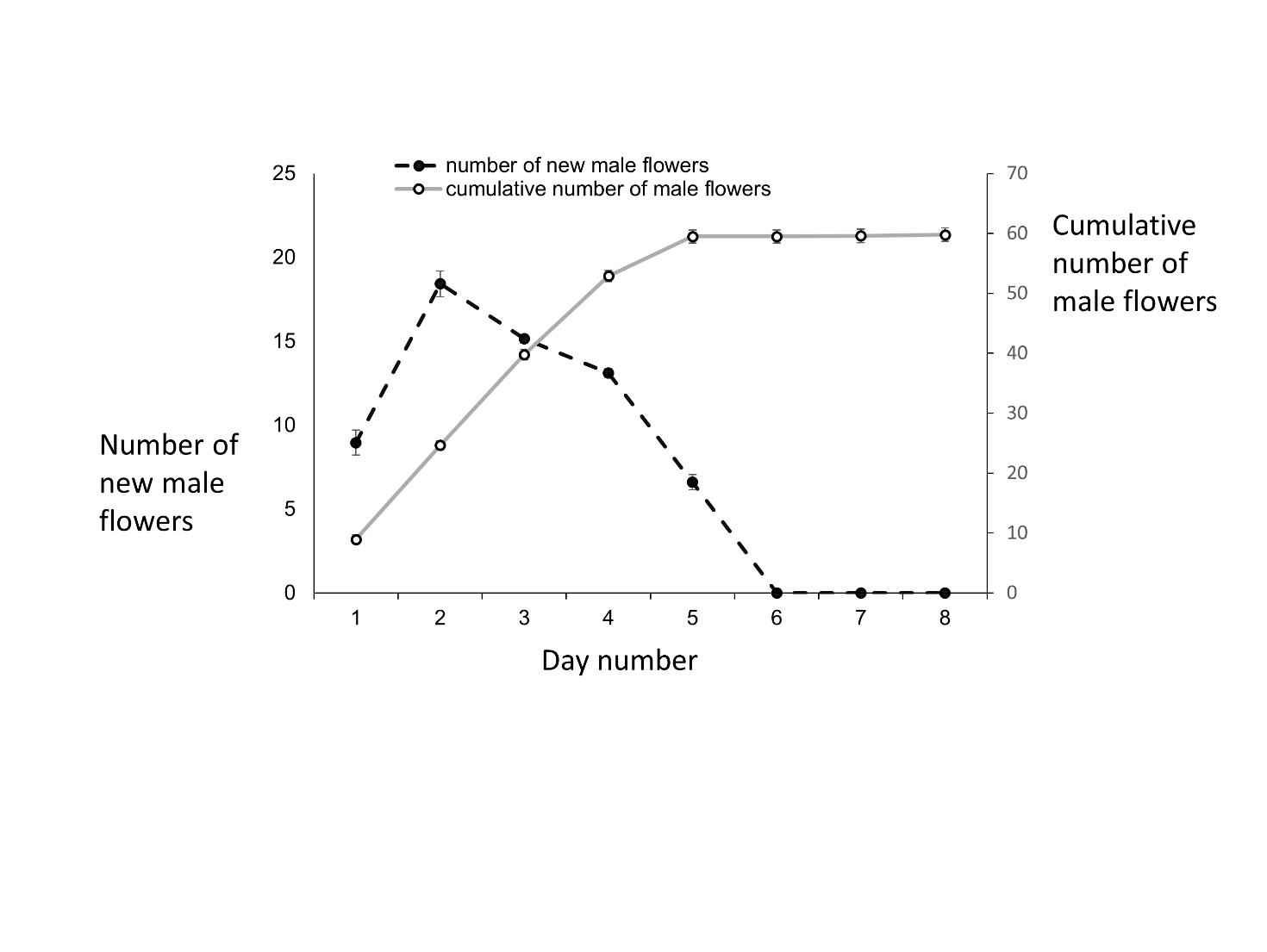


***Figure S2.*** (a) Natural sunlight and (b) Ultraviolet light on flower-heads of *Chrysanthemoides monilifera* ssp. *rotundata*. Images taken with a NIKON D1X. UV images taken with a Baader U-Filter, 60nmHBW/3200380 nm, Nikon Speedlight Flash SB-14 Modified for Ultraviolet. Exposures were manual, shutter 1/1250, F5, ISO800, focal length 105mm, white balance 0, Exposure +/- = 0. Visible light images were taken with a Nikon D1X with a skylight 1A filter, with a standard white light source. Scale bar = 14 mm


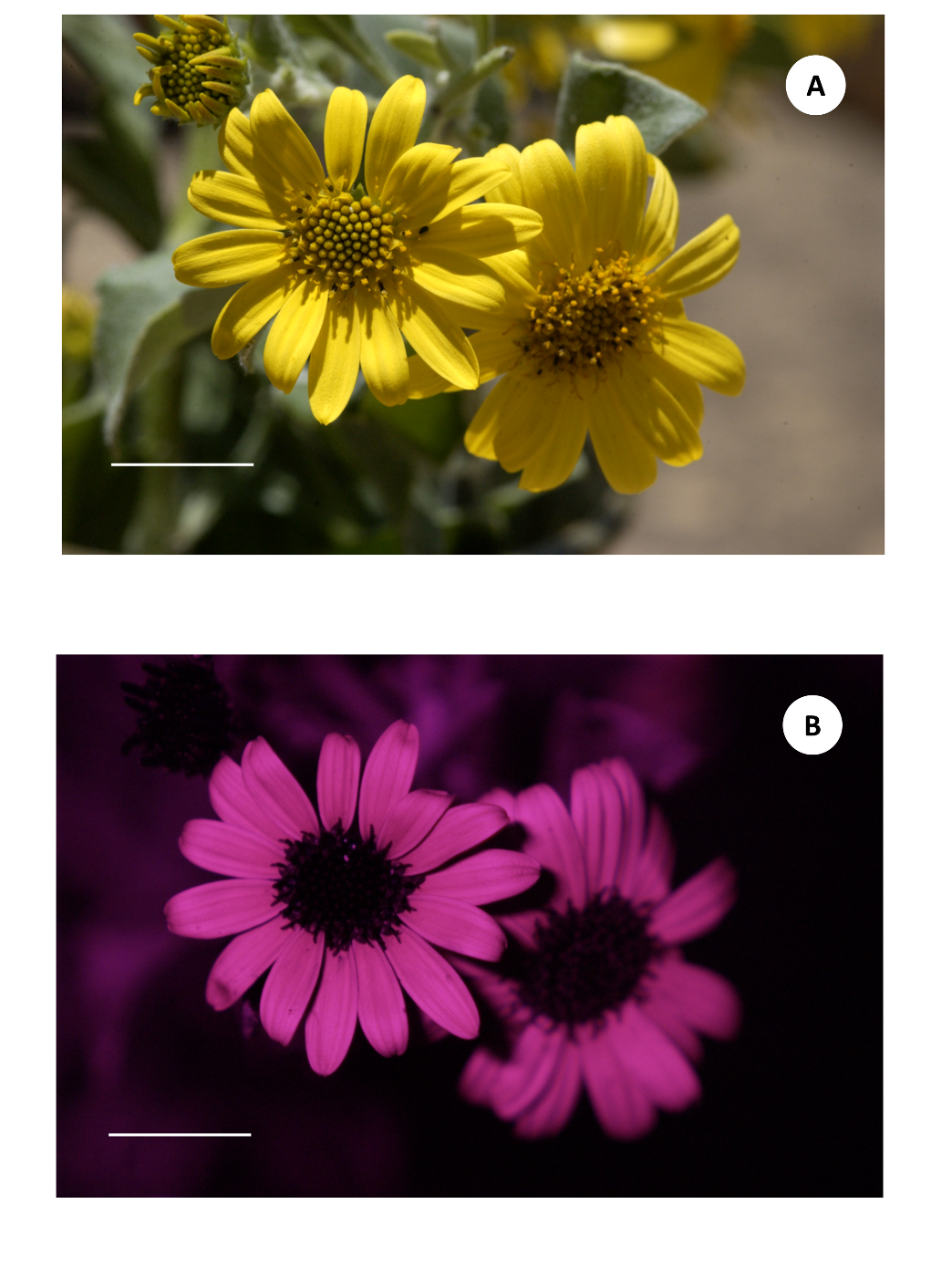


***Figure S3.*** Female flower production and fruit to flower ratios across ten populations for *Chrysanthemoides* *monilifera* ssp. *rotundata*. Results for six populations are from the outcrossing experiments in the glasshouse and the remaining four open populations were open pollinations in the naturalised coastal areas (Hungry Head cabin, Hungry Head dunes Tuckers Rock Sand dunes) or the artificial population comprised of five clones from Arrawarra and grown outside the glasshouse at UNE.


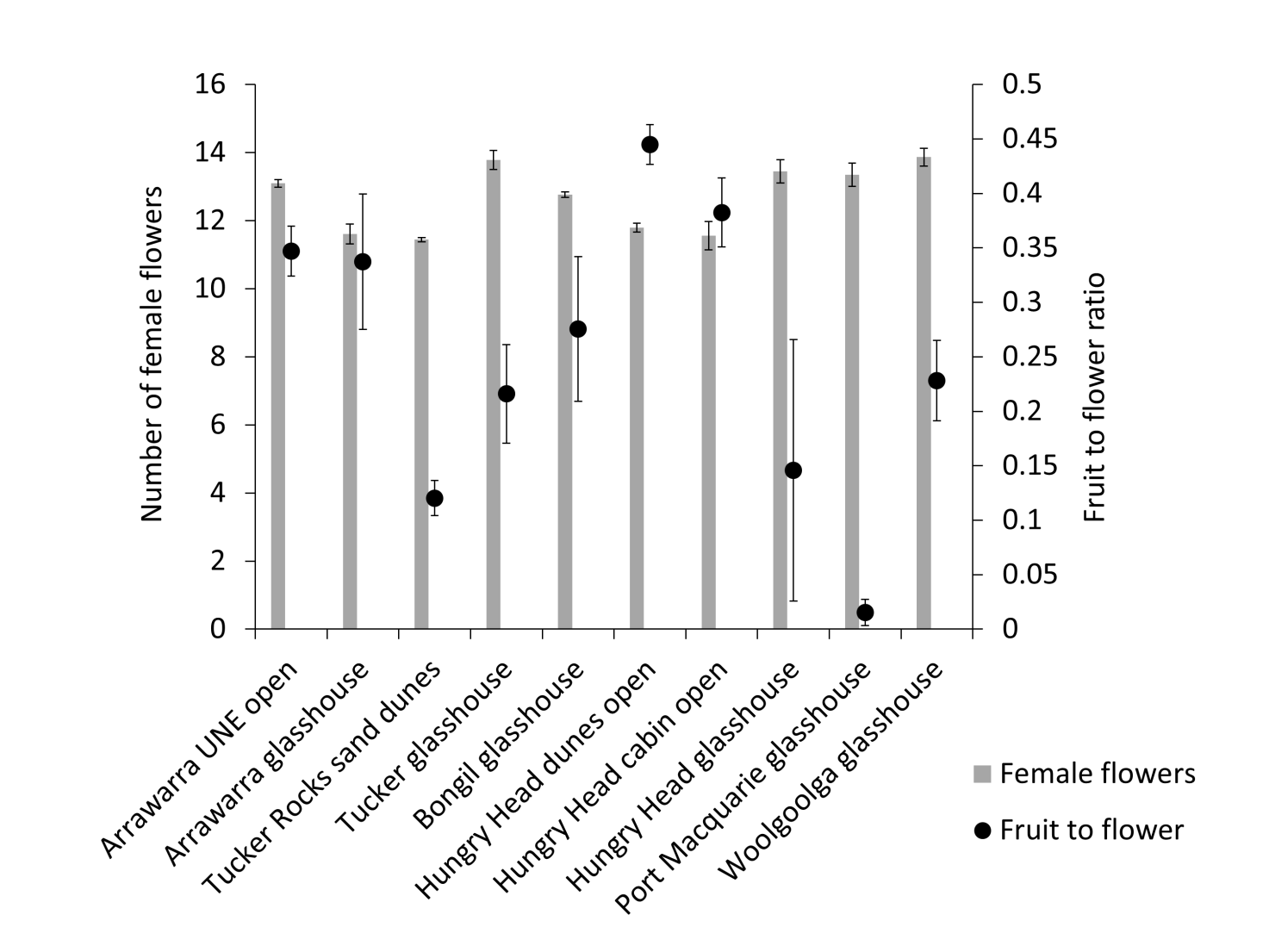
***Table S1. Nectar study - methods and results.***

Individual flower flower-heads from four glasshouse populations (Bongil Bongil, Port Macquarie, Tucker’s Rock and Woolgoolga) were harvested and flowers were separated into male and female florets. A 0.5 μL microcapillary tube (32mm long, Drummond, USA, Cat. No. 1-000-0005) was inserted into the base of each floret to wick the nectar into the tube. Volumes were small so we pooled 10 florets per head to achieve a readable sample. The length of nectar wicked into the tube was measured and the final volume extrapolated against the 32mm length of the 0.5 μL microcapillary tube. We used a Bellingham and Stanley Eclipse Professional Grade Optical Hand Held Refractometer (45-05 Sugar % (Brix) to provide a % sucrose equivalent reading. Some samples were insufficient for a refractometer reading although we had at least 17 samples per population with sufficient nectar. N = number of flower-heads.

We only detected nectar in male florets. Analysis of variance was used on homoscedastic data to determine if significant differences occurred among populations in the volumes of nectar and % sucrose from male florets. No significant differences were detected among populations for nectar volume (*F_3, 156_ = 0.09, P = 0.97*) or nectar concentration (*F_3, 70_ = 2.02, P = 0.12*).

| *Population* | *Volume* μL *± 1SE (N)*  *10 female florets* | *Volume* μL *± 1SE (N)*  *10 male florets* | *Mean % sucrose ± 1SE (N)*  *10 male florets* |
| --- | --- | --- | --- |
| Bongil Bongil | 0 (40) | 0.055*±*0.01 (40) | 58.33*±*1.39 (18) |
| Port Macquarie | 0 (40) | 0.055*±*0.01 (40) | 61.05*±*1.33 (19) |
| Tuckers Rock | 0 (40) | 0.052*±*0.01 (40) | 61.41*±*1.14 (17) |
| Woolgoolga | 0 (40) | 0.060*±*0.01 (40) | 58.25*±*0.88 (20) |
| Total | 0 (160) | 0.055*±*0.01 (160) | 59.72*±*0.61 (74) |

***Table S2.*** Fruit mass (mean ± 1SE mg, *N= number of fruits*) results from the glasshouse breeding system experiments across six populations using two types of pollen donors, from within populations (intra) and from outside populations (inter) and within population pooled data. n/a = intra population pollen was not used due to a shortage of flowers. Fruits from the outcrossed treatments did not vary significantly in their mass among the populations in which we only used intra-population pollen donors (4 populations, *F_3, 279_ = 0.34, P = 0.79*, pooled fruit mass 48.35±0.91 mg, *N= 283*). For populations in which we used both intra and inter-population pollen donors (4 populations), we found a significant interaction between population and pollen source (intra vs inter; interaction *F_3, 453_ = 6.14, P = 0.0004*), due to Arrawarra being the only population with lower fruit mass from inter population outcrosses.

|  | *Fruit mass (mg)* | | |
| --- | --- | --- | --- |
|  | *Donor source* | |  |
| *Population* | *Intra population* | *Inter population* | *Pooled* |
| Arrawarra | 46.42±0.92 (*71*) | 39.51±2.43 (*28*) | 44.47±1.00 (*99*) |
| Bongil Bongil | n/a | 55.52±2.12 (*95*) | 55.52±2.12 (*95*) |
| Hungry Head | 51.62±1.16 (*7*) | 62.92±7.01 (*3*) | 55.01±2.62 (*10*) |
| Port Macquarie | n/a | 48.98±2.63 (*26*) | 48.98±2.63 (*26*) |
| Tucker's Rock | 48.27±3.01 (*41*) | 49.28±2.00 (*99*) | 48.98±1.66 (*140*) |
| Woolgoolga | 49.06±1.32 (*164*) | 60.78±2.46 (*48*) | 51.71±1.21 (*212*) |
| Pooled Populations | 48.35±0.91 (*283*) | 52.30±1.12 (*299*) | 50.38± 0.73 (582) |
